# 2,2,7-Trimethyl-2,3-dihydro­quinazolin-4(1*H*)-one

**DOI:** 10.1107/S1600536809018480

**Published:** 2009-05-20

**Authors:** Ling Zhang, Daxin Shi, Yanqiu Fan, Dongfeng Qian, Jiarong Li

**Affiliations:** a School of Chemical Engineering and Environment, Beijing Institute of Technology, Beijing 100081, People’s Republic of China

## Abstract

There are two independent mol­ecules in the the asymmetric unit of the title compound, C_11_H_14_N_2_O. The heterocyclic ring of the bicyclic system has a sofa conformation, with the C atom bearing the two methyl groups displaced by 0.541 (7) Å from the rest of the atoms of the ring [planar to within 0.064 (9) Å]. Mol­ecules are linked into centrosymmetric dimers *via* N—H⋯O hydrogen bonds.

## Related literature

For medicinal and biological properties of dihydro­quinazolin-4(3*H*)-one derivatives, see: Jackson *et al.* (2007 ▶); Shi *et al.* (2004 ▶). For a related structure, see: Zhang *et al.* (2008 ▶).
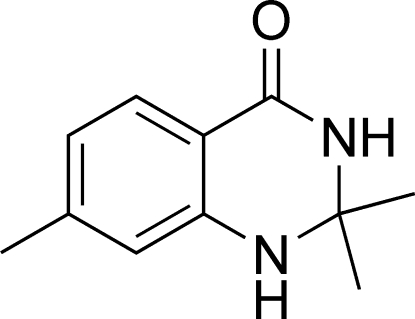


## Experimental

### 

#### Crystal data

C_11_H_14_N_2_O
*M* *_r_* = 190.24
Orthorhombic, 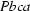

*a* = 19.538 (4) Å
*b* = 10.104 (2) Å
*c* = 20.735 (4) Å
*V* = 4093.4 (14) Å^3^
*Z* = 16
Mo *K*α radiation
μ = 0.08 mm^−1^
*T* = 113 K
0.18 × 0.16 × 0.12 mm

#### Data collection

Rigaku Saturn diffractometer
Absorption correction: multi-scan (*CrystalClear*; Rigaku/MSC, 2005 ▶) *T*_min_ = 0.986, *T*_max_ = 0.990
31345 measured reflections
3599 independent reflections
3269 reflections with *I* > 2σ(*I*)
*R*_int_ = 0.037

#### Refinement

*R*[*F* ^2^ > 2σ(*F* ^2^)] = 0.043
*wR*(*F* ^2^) = 0.119
*S* = 1.07
3599 reflections
274 parameters
4 restraints
H atoms treated by a mixture of independent and constrained refinement
Δρ_max_ = 0.26 e Å^−3^
Δρ_min_ = −0.27 e Å^−3^

### 

Data collection: *CrystalClear* (Rigaku/MSC, 2005 ▶); cell refinement: *CrystalClear*; data reduction: *CrystalClear*; program(s) used to solve structure: *SHELXS97* (Sheldrick, 2008 ▶); program(s) used to refine structure: *SHELXL97* (Sheldrick, 2008 ▶); molecular graphics: *SHELXTL* (Sheldrick, 2008 ▶); software used to prepare material for publication: *SHELXTL*.

## Supplementary Material

Crystal structure: contains datablocks global, I. DOI: 10.1107/S1600536809018480/jh2079sup1.cif

Structure factors: contains datablocks I. DOI: 10.1107/S1600536809018480/jh2079Isup2.hkl

Additional supplementary materials:  crystallographic information; 3D view; checkCIF report

## Figures and Tables

**Table 1:** Hydrogen-bond geometry (Å, °)

*D*—H⋯*A*	*D*—H	H⋯*A*	*D*⋯*A*	*D*—H⋯*A*
N1—H1⋯O1^i^	0.891 (9)	2.221 (10)	3.0917 (16)	165.7 (14)
N2—H2⋯O2	0.901 (9)	2.029 (10)	2.9144 (15)	167.2 (16)
N4—H4⋯O1	0.897 (9)	1.956 (10)	2.8488 (16)	173.3 (18)

Symmetry code: (i) 
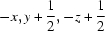
.

**Fig. 1.**
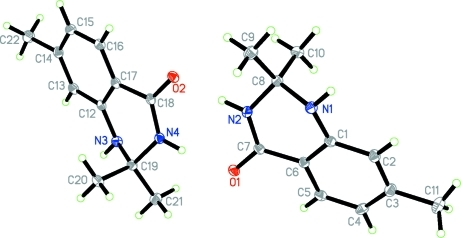
Molecular structure of the title compound with thermal displacement ellipsoids drawn at the 30% probability level.

**Fig. 2.**
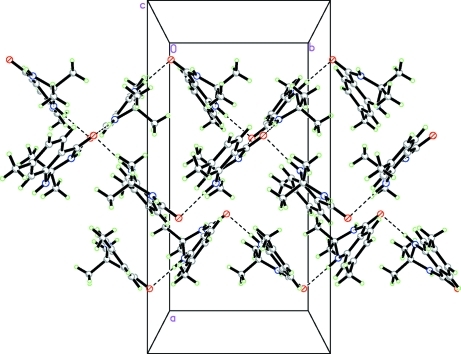
The crystal packing of the title compound, viewed along the c axis

## supplementary crystallographic information

### Comment

Derivatives of dihydroquinazolin-4(3*H*)-one are valuable synthetic intermediates featuring common structural motif found in a variety of compounds with interesting medicinal and biological properties (Shi *et al.*, 2004; Jackson *et al.*, 2007).

In the molecule of the title compound (Fig. 1), the 1,3-diazacyclohexane moiety of the bicyclic system has a sofa conformation with the C8 atom displaced by 0.541 (7) Å from the rest of the atoms of the 1,3-diazacyclohexane ring (planar within 0.064 (9) Å). The dihedral angle between C8, C9, C10 plane and the plane (N1, N2, C8) is 89.8 (3)°.

Molecules in crystal are linked into centrosymmetric dimers *via* N2—H2···O2^i^ bonds (N2—H2 0.901 (9) Å, H2···O2^i^ 2.029 (10) Å, N2—H2···O2^i^ 167.2 (16)°) and N4—H4···O1^i^ bonds (N4—H4 0.897 (9) Å, H4···O1^i^ 1.956 (10) Å, N4—H4···O1^i^ 173.3 (18)°)(Fig. 2).

The molecular geometry and overall crystal structure of the title compound are quite similar to those observed in the structure of its close analog which lacks the methyl substituent in position 6 of the tetrahydroquinalazolinone system (Shi *et al.*, 2003).

### Experimental

A solution of 2-amino-5-methylbenzonitrile (10 mmol) and sodium methylate (10 mmol) in acetone (10 ml), was refluxed for 2 h. The reaction mixture was cooled, to room temperature and poured into 20 ml of water (previously cooled to 20°); then it was extracted with ethyl acetate, distilled off ethyl acetate to give the title compound. The product was recrystallizated from ethanol and ethyl acetate to give colorless crystalline powder. m.p. 539–540 K; IR (KBr): 3300 (N—H), 3036, 2972 (C—H), 1642 (C═O) cm^-1^; ^1H^-NMR (CDCl3, p.p.m.): 1.54 (6H, s), 2.29 (3H, s) 5.89 (1H, s), 6.66 (1H, s), 7.26 (1H, d), 7.78 (1H, d), 8.19 (1H, br). 50 mg of the obtained product was dissolved in ethyl acetate (5 ml) and the solution was kept at room temperature for 4 d to give colorless single crystals.

### Refinement

C—H were included in the riding model approximation with C—H distances 0.95–0.99 Å, and with *U*_iso_(H) = 1.2*U*_eq_(C) or 1.5*U*_eq_(*C*)(methyl). H atoms of NH group were located in difference Fourrier maps with N—H distances 0.891–0.901 Å with *U*_iso_(H) = 1.2*U*_eq_(N).

### Crystal data

**Table:**

C_11_H_14_N_2_O	*D*_x_ = 1.235 Mg m^−^^3^
*M**_r_* = 190.24	Mo *K*α radiation, λ = 0.71073 Å
Orthorhombic, *P**b**c**a*	Cell parameters from 11344 reflections
*a* = 19.538 (4) Å	θ = 2.0–27.9°
*b* = 10.104 (2) Å	µ = 0.08 mm^−^^1^
*c* = 20.735 (4) Å	*T* = 113 K
*V* = 4093.4 (14) Å^3^	Rhombic, colourless
*Z* = 16	0.18 × 0.16 × 0.12 mm
*F*(000) = 1632	

### Data collection

**Table:**

Rigaku Saturn diffractometer	3599 independent reflections
Radiation source: rotating anode	3269 reflections with *I* > 2σ(*I*)
confocal	*R*_int_ = 0.037
ω scans	θ_max_ = 25.0°, θ_min_ = 2.0°
Absorption correction: multi-scan (*CrystalClear*; Rigaku/MSC, 2005)	*h* = −23→20
*T*_min_ = 0.986, *T*_max_ = 0.990	*k* = −12→12
31345 measured reflections	*l* = −24→24

### Refinement

**Table:**

Refinement on *F*^2^	Primary atom site location: structure-invariant direct methods
Least-squares matrix: full	Secondary atom site location: difference Fourier map
*R*[*F*^2^ > 2σ(*F*^2^)] = 0.043	Hydrogen site location: inferred from neighbouring sites
*wR*(*F*^2^) = 0.119	H atoms treated by a mixture of independent and constrained refinement
*S* = 1.07	*w* = 1/[σ^2^(*F*_o_^2^) + (0.0707*P*)^2^ + 1.3149*P*] where *P* = (*F*_o_^2^ + 2*F*_c_^2^)/3
3599 reflections	(Δ/σ)_max_ = 0.001
274 parameters	Δρ_max_ = 0.26 e Å^−^^3^
4 restraints	Δρ_min_ = −0.27 e Å^−^^3^

### Special details

**Table:**

Geometry. All e.s.d.'s (except the e.s.d. in the dihedral angle between two l.s. planes) are estimated using the full covariance matrix. The cell e.s.d.'s are taken into account individually in the estimation of e.s.d.'s in distances, angles and torsion angles; correlations between e.s.d.'s in cell parameters are only used when they are defined by crystal symmetry. An approximate (isotropic) treatment of cell e.s.d.'s is used for estimating e.s.d.'s involving l.s. planes.
Refinement. Refinement of *F*^2^ against ALL reflections. The weighted *R*-factor *wR* and goodness of fit *S* are based on *F*^2^, conventional *R*-factors *R* are based on *F*, with *F* set to zero for negative *F*^2^. The threshold expression of *F*^2^ > σ(*F*^2^) is used only for calculating *R*-factors(gt) *etc*. and is not relevant to the choice of reflections for refinement. *R*-factors based on *F*^2^ are statistically about twice as large as those based on *F*, and *R*- factors based on ALL data will be even larger.

### Fractional atomic coordinates and isotropic or equivalent isotropic displacement parameters (Å^2^)

**Table:**

	*x*	*y*	*z*	*U*_iso_*/*U*_eq_	
O1	0.12627 (5)	0.35620 (10)	0.25627 (5)	0.0229 (2)	
O2	0.12436 (5)	0.41999 (10)	0.08661 (5)	0.0217 (2)	
N1	−0.01899 (6)	0.63561 (12)	0.26515 (5)	0.0207 (3)	
N2	0.05728 (6)	0.50293 (12)	0.20547 (6)	0.0205 (3)	
N3	0.27303 (6)	0.14582 (12)	0.08958 (6)	0.0217 (3)	
N4	0.18129 (6)	0.25660 (11)	0.13865 (6)	0.0198 (3)	
C1	0.01921 (7)	0.61666 (14)	0.32076 (6)	0.0191 (3)	
C2	0.00343 (7)	0.67890 (14)	0.37919 (7)	0.0231 (3)	
H2A	−0.0325	0.7392	0.3807	0.028*	
C3	0.04036 (8)	0.65240 (15)	0.43478 (7)	0.0265 (3)	
C4	0.09473 (8)	0.56177 (16)	0.43222 (7)	0.0283 (3)	
H4A	0.1201	0.5440	0.4692	0.034*	
C5	0.11057 (8)	0.49918 (15)	0.37522 (7)	0.0241 (3)	
H5	0.1464	0.4386	0.3740	0.029*	
C6	0.07354 (7)	0.52554 (13)	0.31920 (6)	0.0189 (3)	
C7	0.08837 (7)	0.45605 (13)	0.25853 (6)	0.0187 (3)	
C8	0.01727 (7)	0.62612 (14)	0.20330 (6)	0.0195 (3)	
C9	−0.03515 (8)	0.61367 (16)	0.14934 (7)	0.0283 (4)	
H9A	−0.0645	0.5394	0.1577	0.043*	
H9B	−0.0119	0.6008	0.1090	0.043*	
H9C	−0.0621	0.6930	0.1473	0.043*	
C10	0.06439 (7)	0.74473 (15)	0.19217 (7)	0.0246 (3)	
H10A	0.0374	0.8237	0.1883	0.037*	
H10B	0.0901	0.7315	0.1533	0.037*	
H10C	0.0952	0.7534	0.2280	0.037*	
C11	0.02288 (9)	0.72142 (19)	0.49722 (7)	0.0377 (4)	
H11A	−0.0050	0.7975	0.4883	0.057*	
H11B	0.0643	0.7490	0.5183	0.057*	
H11C	−0.0017	0.6617	0.5248	0.057*	
C12	0.25326 (7)	0.19204 (14)	0.02964 (6)	0.0197 (3)	
C13	0.28991 (7)	0.16131 (14)	−0.02662 (7)	0.0230 (3)	
H13	0.3286	0.1079	−0.0238	0.028*	
C14	0.26961 (7)	0.20891 (15)	−0.08615 (7)	0.0236 (3)	
C15	0.21162 (7)	0.29057 (15)	−0.09035 (7)	0.0236 (3)	
H15	0.1971	0.3218	−0.1303	0.028*	
C16	0.17618 (7)	0.32435 (14)	−0.03528 (7)	0.0219 (3)	
H16	0.1382	0.3796	−0.0383	0.026*	
C17	0.19642 (7)	0.27684 (14)	0.02505 (7)	0.0194 (3)	
C18	0.16363 (7)	0.32180 (13)	0.08495 (6)	0.0188 (3)	
C19	0.21890 (7)	0.13021 (14)	0.13797 (7)	0.0200 (3)	
C20	0.16986 (8)	0.01709 (14)	0.12152 (7)	0.0253 (3)	
H20A	0.1342	0.0131	0.1533	0.038*	
H20B	0.1945	−0.0651	0.1212	0.038*	
H20C	0.1501	0.0322	0.0798	0.038*	
C21	0.25077 (8)	0.10928 (16)	0.20382 (7)	0.0280 (3)	
H21A	0.2808	0.1818	0.2135	0.042*	
H21B	0.2763	0.0281	0.2038	0.042*	
H21C	0.2153	0.1047	0.2358	0.042*	
C22	0.30798 (8)	0.17113 (17)	−0.14637 (7)	0.0321 (4)	
H22A	0.3335	0.2460	−0.1617	0.048*	
H22B	0.2761	0.1436	−0.1789	0.048*	
H22C	0.3388	0.0998	−0.1369	0.048*	
H1	−0.0488 (7)	0.7022 (12)	0.2664 (8)	0.024 (4)*	
H2	0.0715 (9)	0.4736 (18)	0.1667 (6)	0.037 (5)*	
H3	0.3037 (7)	0.0800 (13)	0.0885 (8)	0.029 (4)*	
H4	0.1643 (9)	0.2814 (18)	0.1770 (6)	0.039 (5)*	

### Atomic displacement parameters (Å^2^)

**Table:**

	*U*^11^	*U*^22^	*U*^33^	*U*^12^	*U*^13^	*U*^23^
O1	0.0258 (5)	0.0182 (5)	0.0248 (5)	0.0048 (4)	0.0029 (4)	0.0006 (4)
O2	0.0206 (5)	0.0185 (5)	0.0261 (5)	0.0038 (4)	0.0035 (4)	0.0001 (4)
N1	0.0172 (6)	0.0233 (7)	0.0214 (6)	0.0043 (5)	0.0014 (5)	0.0003 (5)
N2	0.0229 (6)	0.0203 (6)	0.0182 (6)	0.0025 (5)	0.0011 (5)	−0.0018 (5)
N3	0.0178 (6)	0.0224 (7)	0.0247 (6)	0.0043 (5)	0.0010 (5)	0.0004 (5)
N4	0.0205 (6)	0.0187 (6)	0.0201 (6)	0.0014 (5)	0.0013 (5)	−0.0017 (5)
C1	0.0174 (7)	0.0181 (7)	0.0217 (7)	−0.0025 (5)	0.0015 (5)	0.0020 (5)
C2	0.0211 (7)	0.0227 (8)	0.0256 (7)	0.0006 (6)	0.0037 (6)	−0.0015 (6)
C3	0.0291 (8)	0.0280 (8)	0.0223 (7)	−0.0032 (6)	0.0042 (6)	−0.0024 (6)
C4	0.0337 (8)	0.0324 (9)	0.0189 (7)	0.0014 (7)	−0.0050 (6)	0.0035 (6)
C5	0.0257 (8)	0.0207 (7)	0.0259 (8)	0.0026 (6)	−0.0009 (6)	0.0037 (6)
C6	0.0185 (7)	0.0166 (7)	0.0215 (7)	−0.0018 (5)	0.0016 (5)	0.0004 (5)
C7	0.0168 (7)	0.0162 (7)	0.0229 (7)	−0.0033 (5)	0.0022 (5)	0.0012 (5)
C8	0.0184 (7)	0.0209 (7)	0.0193 (7)	0.0026 (5)	−0.0001 (5)	0.0005 (5)
C9	0.0255 (8)	0.0347 (9)	0.0247 (8)	0.0010 (6)	−0.0044 (6)	0.0001 (6)
C10	0.0230 (7)	0.0237 (8)	0.0272 (7)	0.0014 (6)	0.0020 (6)	0.0038 (6)
C11	0.0422 (10)	0.0467 (11)	0.0241 (8)	0.0014 (8)	0.0030 (7)	−0.0076 (7)
C12	0.0191 (7)	0.0158 (7)	0.0244 (7)	−0.0028 (5)	−0.0003 (6)	−0.0020 (5)
C13	0.0203 (7)	0.0200 (7)	0.0288 (8)	0.0015 (6)	0.0037 (6)	−0.0019 (6)
C14	0.0253 (7)	0.0202 (7)	0.0252 (7)	−0.0048 (6)	0.0048 (6)	−0.0022 (6)
C15	0.0266 (7)	0.0222 (8)	0.0221 (7)	−0.0032 (6)	−0.0025 (6)	0.0013 (6)
C16	0.0207 (7)	0.0184 (7)	0.0265 (7)	−0.0008 (5)	−0.0008 (6)	−0.0002 (6)
C17	0.0179 (7)	0.0169 (7)	0.0233 (7)	−0.0018 (5)	0.0004 (5)	−0.0020 (5)
C18	0.0161 (7)	0.0163 (7)	0.0241 (7)	−0.0040 (5)	0.0000 (5)	−0.0022 (6)
C19	0.0184 (7)	0.0184 (7)	0.0232 (7)	0.0028 (5)	0.0019 (5)	−0.0001 (6)
C20	0.0255 (8)	0.0193 (8)	0.0312 (8)	0.0002 (6)	0.0022 (6)	0.0001 (6)
C21	0.0273 (8)	0.0324 (9)	0.0244 (7)	0.0058 (6)	−0.0008 (6)	0.0008 (6)
C22	0.0357 (9)	0.0330 (9)	0.0278 (8)	0.0030 (7)	0.0087 (7)	−0.0009 (7)

### Geometric parameters (Å, °)

**Table:**

O1—C7	1.2523 (17)	C9—H9C	0.9600
O2—C18	1.2548 (17)	C10—H10A	0.9600
N1—C1	1.3870 (18)	C10—H10B	0.9600
N1—C8	1.4681 (17)	C10—H10C	0.9600
N1—H1	0.891 (9)	C11—H11A	0.9600
N2—C7	1.3430 (18)	C11—H11B	0.9600
N2—C8	1.4706 (17)	C11—H11C	0.9600
N2—H2	0.901 (9)	C12—C13	1.403 (2)
N3—C12	1.3829 (18)	C12—C17	1.406 (2)
N3—C19	1.4662 (18)	C13—C14	1.383 (2)
N3—H3	0.896 (9)	C13—H13	0.9300
N4—C18	1.3390 (18)	C14—C15	1.404 (2)
N4—C19	1.4734 (17)	C14—C22	1.506 (2)
N4—H4	0.897 (9)	C15—C16	1.378 (2)
C1—C2	1.399 (2)	C15—H15	0.9300
C1—C6	1.4056 (19)	C16—C17	1.397 (2)
C2—C3	1.386 (2)	C16—H16	0.9300
C2—H2A	0.9300	C17—C18	1.4695 (19)
C3—C4	1.404 (2)	C19—C21	1.515 (2)
C3—C11	1.510 (2)	C19—C20	1.530 (2)
C4—C5	1.376 (2)	C20—H20A	0.9600
C4—H4A	0.9300	C20—H20B	0.9600
C5—C6	1.394 (2)	C20—H20C	0.9600
C5—H5	0.9300	C21—H21A	0.9600
C6—C7	1.4694 (19)	C21—H21B	0.9600
C8—C9	1.5220 (19)	C21—H21C	0.9600
C8—C10	1.5288 (19)	C22—H22A	0.9600
C9—H9A	0.9600	C22—H22B	0.9600
C9—H9B	0.9600	C22—H22C	0.9600
			
C1—N1—C8	117.23 (11)	C3—C11—H11A	109.5
C1—N1—H1	115.6 (10)	C3—C11—H11B	109.5
C8—N1—H1	113.0 (10)	H11A—C11—H11B	109.5
C7—N2—C8	124.33 (11)	C3—C11—H11C	109.5
C7—N2—H2	118.4 (11)	H11A—C11—H11C	109.5
C8—N2—H2	114.6 (12)	H11B—C11—H11C	109.5
C12—N3—C19	116.74 (11)	N3—C12—C13	122.00 (13)
C12—N3—H3	114.5 (11)	N3—C12—C17	119.16 (12)
C19—N3—H3	114.9 (11)	C13—C12—C17	118.79 (13)
C18—N4—C19	123.15 (11)	C14—C13—C12	121.23 (13)
C18—N4—H4	120.3 (12)	C14—C13—H13	119.4
C19—N4—H4	115.8 (12)	C12—C13—H13	119.4
N1—C1—C2	122.63 (12)	C13—C14—C15	119.41 (13)
N1—C1—C6	118.53 (12)	C13—C14—C22	120.61 (14)
C2—C1—C6	118.74 (13)	C15—C14—C22	119.96 (13)
C3—C2—C1	121.23 (13)	C16—C15—C14	119.97 (13)
C3—C2—H2A	119.4	C16—C15—H15	120.0
C1—C2—H2A	119.4	C14—C15—H15	120.0
C2—C3—C4	119.25 (13)	C15—C16—C17	120.97 (13)
C2—C3—C11	120.41 (14)	C15—C16—H16	119.5
C4—C3—C11	120.34 (14)	C17—C16—H16	119.5
C5—C4—C3	120.19 (13)	C16—C17—C12	119.58 (13)
C5—C4—H4A	119.9	C16—C17—C18	121.82 (12)
C3—C4—H4A	119.9	C12—C17—C18	118.39 (12)
C4—C5—C6	120.74 (14)	O2—C18—N4	121.59 (12)
C4—C5—H5	119.6	O2—C18—C17	122.29 (12)
C6—C5—H5	119.6	N4—C18—C17	116.01 (12)
C5—C6—C1	119.85 (13)	N3—C19—N4	105.84 (11)
C5—C6—C7	121.31 (12)	N3—C19—C21	109.58 (11)
C1—C6—C7	118.79 (12)	N4—C19—C21	108.49 (11)
O1—C7—N2	121.39 (12)	N3—C19—C20	112.31 (11)
O1—C7—C6	122.25 (12)	N4—C19—C20	109.72 (11)
N2—C7—C6	116.33 (12)	C21—C19—C20	110.72 (12)
N1—C8—N2	106.56 (11)	C19—C20—H20A	109.5
N1—C8—C9	108.83 (11)	C19—C20—H20B	109.5
N2—C8—C9	108.07 (11)	H20A—C20—H20B	109.5
N1—C8—C10	111.79 (11)	C19—C20—H20C	109.5
N2—C8—C10	110.37 (11)	H20A—C20—H20C	109.5
C9—C8—C10	111.04 (12)	H20B—C20—H20C	109.5
C8—C9—H9A	109.5	C19—C21—H21A	109.5
C8—C9—H9B	109.5	C19—C21—H21B	109.5
H9A—C9—H9B	109.5	H21A—C21—H21B	109.5
C8—C9—H9C	109.5	C19—C21—H21C	109.5
H9A—C9—H9C	109.5	H21A—C21—H21C	109.5
H9B—C9—H9C	109.5	H21B—C21—H21C	109.5
C8—C10—H10A	109.5	C14—C22—H22A	109.5
C8—C10—H10B	109.5	C14—C22—H22B	109.5
H10A—C10—H10B	109.5	H22A—C22—H22B	109.5
C8—C10—H10C	109.5	C14—C22—H22C	109.5
H10A—C10—H10C	109.5	H22A—C22—H22C	109.5
H10B—C10—H10C	109.5	H22B—C22—H22C	109.5
			
C8—N1—C1—C2	149.48 (13)	C19—N3—C12—C13	−152.94 (13)
C8—N1—C1—C6	−34.23 (18)	C19—N3—C12—C17	29.74 (18)
N1—C1—C2—C3	176.45 (13)	N3—C12—C13—C14	−179.69 (13)
C6—C1—C2—C3	0.2 (2)	C17—C12—C13—C14	−2.4 (2)
C1—C2—C3—C4	0.2 (2)	C12—C13—C14—C15	0.6 (2)
C1—C2—C3—C11	179.50 (14)	C12—C13—C14—C22	−177.81 (13)
C2—C3—C4—C5	−0.7 (2)	C13—C14—C15—C16	1.1 (2)
C11—C3—C4—C5	−179.92 (15)	C22—C14—C15—C16	179.51 (13)
C3—C4—C5—C6	0.7 (2)	C14—C15—C16—C17	−1.0 (2)
C4—C5—C6—C1	−0.2 (2)	C15—C16—C17—C12	−0.8 (2)
C4—C5—C6—C7	−177.87 (13)	C15—C16—C17—C18	173.90 (13)
N1—C1—C6—C5	−176.62 (12)	N3—C12—C17—C16	179.82 (12)
C2—C1—C6—C5	−0.2 (2)	C13—C12—C17—C16	2.4 (2)
N1—C1—C6—C7	1.08 (19)	N3—C12—C17—C18	4.97 (19)
C2—C1—C6—C7	177.51 (12)	C13—C12—C17—C18	−172.44 (12)
C8—N2—C7—O1	−174.19 (12)	C19—N4—C18—O2	170.86 (12)
C8—N2—C7—C6	7.79 (19)	C19—N4—C18—C17	−12.96 (18)
C5—C6—C7—O1	12.0 (2)	C16—C17—C18—O2	−12.3 (2)
C1—C6—C7—O1	−165.67 (12)	C12—C17—C18—O2	162.46 (12)
C5—C6—C7—N2	−170.01 (13)	C16—C17—C18—N4	171.59 (12)
C1—C6—C7—N2	12.33 (18)	C12—C17—C18—N4	−13.69 (18)
C1—N1—C8—N2	49.04 (15)	C12—N3—C19—N4	−50.53 (15)
C1—N1—C8—C9	165.35 (12)	C12—N3—C19—C21	−167.33 (12)
C1—N1—C8—C10	−71.63 (15)	C12—N3—C19—C20	69.18 (16)
C7—N2—C8—N1	−36.65 (16)	C18—N4—C19—N3	43.38 (16)
C7—N2—C8—C9	−153.48 (13)	C18—N4—C19—C21	160.91 (12)
C7—N2—C8—C10	84.92 (15)	C18—N4—C19—C20	−78.01 (15)

### Hydrogen-bond geometry (Å, °)

**Table:**

*D*—H···*A*	*D*—H	H···*A*	*D*···*A*	*D*—H···*A*
N1—H1···O1^i^	0.89 (1)	2.22 (1)	3.0917 (16)	166 (1)
N2—H2···O2	0.90 (1)	2.03 (1)	2.9144 (15)	167 (2)
N4—H4···O1	0.90 (1)	1.96 (1)	2.8488 (16)	173 (2)

Symmetry codes: (i) −*x*, *y*+1/2, −*z*+1/2.
